# Phase-of-firing information coding in laminar cortical architecture

**DOI:** 10.1186/1471-2202-12-S1-P369

**Published:** 2011-07-18

**Authors:** Gleb Basalyga, Marcelo Montemurro, Thomas Wennekers

**Affiliations:** 1 Centre for Robotics and Neural Systems (CRNS), University of Plymouth, PL4 8AA, UK; 2 Faculty of Life Sciences, University of Manchester, M13 9PT, UK

## 

We applied recently developed information theory methods [1,2] to the analysis of cortical responses in a large-scale computational model of cat primary visual cortex [3]. These methods quantify the information conveyed by spikes and by local field potentials (LFPs) in a very general way, without ad hoc assumptions about precisely which stimulus features (orientation, direction, etc.) drive the neuronal response. The phase-of-firing information is the extra information obtained by labeling spikes with the value of the LFP phase [2]. In order to gain insight into the information-processing properties of laminar cortical microcircuits, we calculated the spike count information conveyed by firing rates and the phase-of-firing information conveyed by LFPs for each layer of primary visual cortex.

**Figure 1:**
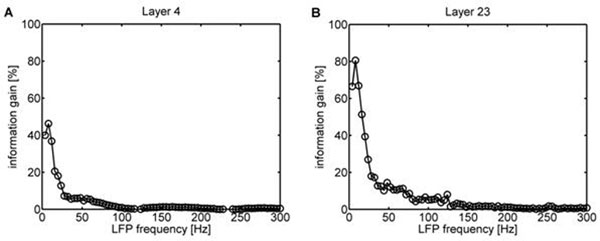
The phase-of-firing information as function of the considered LFP frequency (computed up to 300 Hz). The circles denote the information gain by the phase-of-firing code compared to the spike count code for Layer 4 (A) and for Layer 2/3 (B).

We found that there is substantially more information in the phase code compared with the spike rate alone for low LFP frequencies (< 30 Hz). Figure 1 shows that the information gain for the phase code may reach 80 % in Layer 2/3, while in Layer 4 it reaches only 40 %, compared to the spike count code. These data support the hypothesis that the thalamo-cortical layers, which receive direct sensory input, may rely more on spikes to convey the information, while the cortico-cortical layers with strong recurrent connectivity may use the phase code and LFP signals for information coding.
